# Constructed Wetlands with Novel Substrate Exposed to Nano-Plastics: Mitigating the Effects of Substrate Enzyme and Ecological Processes

**DOI:** 10.3390/toxics13090800

**Published:** 2025-09-20

**Authors:** Luming Wang, Juan Huang, Jing Tuo, Jin Xu, Xinwei Li

**Affiliations:** 1 Department of Municipal Engineering, School of Civil Engineering, Southeast University, Nanjing 210096, China; 230228328@seu.edu.cn (L.W.); tjyx914@163.com (J.T.); 220211267@seu.edu.cn (X.L.); 2 Jiangxi Province Key Laboratory of Watershed Ecological Process and Information, Jiujiang 332005, China; 3 School of Environmental Engineering, Nanjing Institute of Technology, Nanjing 211167, China

**Keywords:** constructed wetland, nano-plastics, MBF (modified basalt fiber), substrate enzyme, microbial community

## Abstract

The widespread occurrence of nano-plastics (NPs) in aquatic environments poses emerging challenges to the pollutant removal performance and ecological stability of constructed wetlands (CWs). This study investigates the performance of calcium-modified (Ca-MBF) and manganese-modified basalt fiber (Mn-MBF) bio-nests as novel substrates to mitigate NP-induced inhibition of CWs. Laboratory-scale CWs were operated for 180 days to evaluate substrate-associated enzyme activities, microbial community structure, and functional gene profiles. Results showed that Mn-MBF bio-nests enhanced the activities of dehydrogenase (DHA), urease (UR), ammonia monooxygenase (AMO), nitrite oxidoreductase (NOR), nitrate reductase (NAR), nitrite reductase (NIR), and phosphatase (PST) by 86.2%, 65.5%, 127.0%, 62.8%, 131.5%, 65.3%, and 107.0%, respectively, compared with the control. In contrast, Ca-MBF bio-nests increased these enzyme activities by 48.6%, 53.5%, 67.0%, 30.6%, 95.0%, 45.3%, and 54.6%, respectively. MBF bio-nests also enhanced microbial diversity, enriched denitrifying and phosphorus-removing bacteria (e.g., Thauera, Plasticicumulans), and promoted extracellular polymeric substance secretion. Functional gene prediction indicated elevated abundances of nitrogen cycle-related genes, thereby enhancing nitrification, denitrification, and phosphorus removal processes. These synergistic effects collectively improved nitrification, denitrification, and phosphorus removal efficiency, with Mn-MBF showing superior performance. This study highlights MBF bio-nests as a sustainable strategy to enhance the resilience and long-term operational stability of CWs in environments impacted by nano-plastic pollution.

## 1. Introduction

Agricultural and industrial activities frequently contribute to the eutrophication of lakes and rivers by discharging various pollutants into aquatic environments [1,2,3]. Constructed wetlands (CWs) have been globally recognized as a sustainable and cost-effective approach for pollution mitigation [4]. The nutrient removal efficiency of CWs primarily depends on substrate adsorption, plant uptake, and microbial metabolism [5,6]. Substrates not only serve as habitats for microorganisms but also supply essential nutrients and electron donors required for microbial metabolic processes [7]. Moreover, the enzymatic activity and metabolic functions of substrates actively participate in pollutant degradation [8]. Consequently, the selection of suitable substrates is crucial for enhancing nitrogen removal efficiency in constructed wetlands.

As fundamental components of CWs, substrate materials serve not only as a pivotal agent for the physical adsorption of pollutants but also as essential biological interfaces supporting plant root colonization and microbial community proliferation [9]. Notably, even when substrate chemical compositions are identical, variations in packing structure can significantly influence microbial community composition and function within CWs [10]. Therefore, the scientific selection of substrate materials and optimization of packing configuration are crucial for maintaining long-term and stable pollutant removal performance. To address the inherent limitations of traditional substrates, various novel functional materials have been introduced into CWs. Traditional wetland substrates (e.g., sand, gravel) face several limitations in practical applications. Their limited surface area results in low pollutant adsorption and rapid saturation. In addition, their simple structure and surface properties provide little support for microbial colonization or functional activity, thereby constraining biodegradation efficiency [11]. Furthermore, these substrates generally lack catalytic activity and electron-transfer capacity, making it difficult to facilitate elemental cycling (e.g., sulfur, iron, manganese) and related pollutant transformation, which ultimately restricts system performance and stability [12]. Basalt fiber (BF), an inorganic material primarily composed of SiO_2_, Al_2_O_3_, Fe_2_O_3_, and CaO [10,13], has emerged as a promising candidate due to its environmental compatibility, high specific surface area, and superior mechanical strength [14,15]. To enhance the pollutant adsorption capacity and bio-synergistic properties of BF, modification strategies such as surface coating (e.g., metal oxide deposition), chemical grafting (functional group modification), and liquid-phase deposition (nanocomposite loading) have been widely adopted [16]. Among these, the incorporation of transition metal ions such as Ca^2+^ and Mn^2+^ has demonstrated synergistic benefits in improving both adsorption efficiency and biological activity [17].

Experimental evidence demonstrates that CWs incorporating calcium-modified basalt fiber (Ca-MBF) exhibit 23–37% higher removal efficiencies for COD and NH_4_^+^-N compared to unmodified BF-based systems, alongside a >40% improvement in operational stability [18]. Furthermore, the presence of Ca^2+^ facilitates the selective enrichment of functional bacterial taxa, including *Nitrosomonas* and *Denitratisoma*, thereby achieving total nitrogen (TN) and total phosphorus (TP) removal rates of 66.2–74.5% and 37.1–42.3%, respectively [12]. Moreover, Mn^2+^ functions as an electron shuttle, accelerating nitrate reduction and enhancing denitrification efficiency by 28–33% [19]. In general, modified basalt fiber (MBF) enhances the electrochemical properties of the substrate surface by participating in the activation of microbial intracellular enzymes (e.g., dehydrogenases) and redox chain transfer and significantly promotes the synthesis of extracellular polymeric substances (EPS) [20]. Functionalized MBF efficiently enriches activated sludge microorganisms due to its three-dimensional network structure, hydrophilic surface, and multi-scale pore distribution [21]. Under hydrodynamic conditions, microbial aggregates self-assemble on the surface of MBFs to form spherical “bio-nests,” within which dissolved oxygen (DO) gradients naturally establish stratified anaerobic, hypoxic, and aerobic microenvironments. This hierarchical microenvironment facilitates the stepwise degradation of pollutants by multiple specialized microorganisms. Recent studies have reported that MBF bio-nests can achieve 81–89% removal of perfluoroalkyl substances (PFAS) through combined adsorption and biodegradation processes [22]. Nevertheless, the transformation pathways and removal mechanisms of MBF bio-nests for emerging contaminants, such as antibiotic resistance genes (ARGs), microplastics (MPs), and pharmaceutically active compounds (PhACs), require further investigation through integrated multi-omics approaches.

The extensive use of polymeric materials has resulted in the continuous emergence of emerging pollutants, notably microplastics (MPs, 1 μm–5 mm) and nanoplastics (NPs, <1 μm), which have become pervasive and persistent contaminants in diverse environmental media [23,24,25]. Due to their exceptionally high specific surface area (>200 m^2^ g^−1^) and surface reactivity, NPs readily adsorb environmental toxins (e.g., polycyclic aromatic hydrocarbons, heavy metals), forming complex pollutants that significantly enhance their ecotoxicity and pose multifaceted threats to global ecosystem health [26,27,28,29]. Migration studies have demonstrated that NPs (1.2–8.7 μm) can enter water treatment systems via surface runoff with an annual flux of 4.3–9.8 kg (km^2^)^−1^, domestic wastewater (10^3^–10^5^ particles L^−1^), and atmospheric deposition [30].

As emerging pollutants, nanoplastics (NPs) progressively accumulate in CWs via surface runoff and sewage infiltration [31]. Their core toxicological mechanisms microbially manifest as a triple cascade effect. Firstly, NPs inhibit substrate enzyme activity, which enrichment on sandy matrices (attachment rate > 97%) markedly suppresses dehydrogenase and urease activities [32], thereby impeding carbohydrate metabolism and nitrogen transformation processes. Consequently, the inhibition translates to reductions of approximately 11.96% and 22.82% in COD and TN removal efficiencies, respectively [33]. Secondly, PS-NPs disrupt microbial community composition, significantly reducing the relative abundance and activity of key nitrifiers (e.g., Nitrosomonas, Nitrospira) and denitrifiers (e.g., Thauera, Zoogloea), which directly contributes to pronounced deterioration in nitrogen removal performance [34]. Third, metabolomic profiling quantified 548 metabolites and identified 291 with altered concentrations following NP exposure. Core metabolic pathways, particularly the tricarboxylic acid (TCA) cycle and amino acid metabolism, were markedly perturbed. Citric acid, threonine, and adenine exhibited decreased concentrations, while amino acids such as serine, phenylalanine, and histidine initially accumulated but subsequently declined with increasing PS-NP exposure [35].

Therefore, Ca-MBF and Mn-MBF bio-nests were used as substrates in constructed wetlands (CWs) to investigate enzyme dynamics and microbial functional activities. While prior studies mainly emphasized pollutant removal efficiency, the microbiological and enzymatic mechanisms—particularly the relationships among microbial gene functions, enzyme activity, and system performance—remain underexplored. To address this gap, this study aimed to: (i) assess substrate-related enzyme activities linked to water quality regulation and plant activity; (ii) characterize microbial community diversity and structure; and (iii) clarify the roles of functional microorganisms and genes in pollutant removal. This work advances mechanistic understanding of how modified basalt fibers enhance microbial ecological functions, providing insights for enzyme- and microbiome-driven wastewater treatment strategies in engineered wetlands.

## 2. Materials and Methods

### 2.1. Cultivation of Modified Basalt Fiber (MBF) and Bio-Nests

This basalt fiber (BF) consists of multi-stranded and single-stranded parallel filaments in an untwisted form (Jiangsu Lugu New Material Technology Co., Ltd., Nanjing, China). To enhance the bio-affinity of basalt fiber (BF), its surface was modified through calcium (Ca) and manganese (Mn) coatings. Prior to coating, BF underwent ultrasonic cleaning in acetone for 2 h to remove surface impurities. It was then treated in 1 mol L^−1^ NaOH at 40 °C for 1 h to induce alkaline etching and enlarge the surface area. Subsequently, BF was immersed in 9.8 mol L^−1^ H_2_O_2_ at 90 °C for 1 h to activate the surface and generate silanol (Si–OH) groups. The resulting material, designated Pretreated Basalt Fiber (PBF), was thoroughly rinsed and dried with deionized water. A schematic of the modification procedures is provided in the Supporting Information, with further details shown in Figure S1.

Both BF and MBF fiber bundles were cut into segments measuring 120 ± 5 mm in length and weighing 15 ± 0.1 g. These segments were subsequently woven into umbrella-shaped structures to form bio-nests. The bio-nests were cultured using the rapid sludge discharge method, with inoculated sludge sourced from a municipal wastewater treatment plant in Nanjing, China (MLSS = 5200 ± 50 mg L^−1^). Throughout the incubation period, dissolved oxygen concentrations were maintained at 2–3 mg L^−1^. The influent was carefully formulated with anhydrous sodium acetate as the carbon source, urea, ammonium sulfate, and potassium nitrate as nitrogen sources, and potassium dihydrogen phosphate as the phosphorus source. Trace elements, including iron, manganese, zinc, and copper, were also supplemented. The influent composition was maintained at a COD:N:P ratio of 100:5:1. Detailed influent parameters during the bio-nest cultivation stage are provided in Table S1 and Figure S2. The cultivation lasted 30 days to ensure structural stability of the bio-nests. The surface morphology and microstructure of each MBF were subsequently characterized using scanning electron microscopy (SEM, FEI-F50, FEI Company, Shanghai, China).

### 2.2. Setup of the Constructed Wetland

Three vertical flow constructed wetland (VFCW) units were established from plexiglass cylinders, each measuring 15 cm in diameter and 50 cm in height. The systems were established on August 30th in Nanjing, Jiangsu Province. The substrate configuration comprised a 30 cm upper layer of river sand (1–2 mm particle size) over a 10 cm lower layer of gravel (5–8 mm particle size). To simulate dark, anoxic conditions and suppress algal growth, the external surfaces of the units were wrapped in black tape.

**Figure 1:**
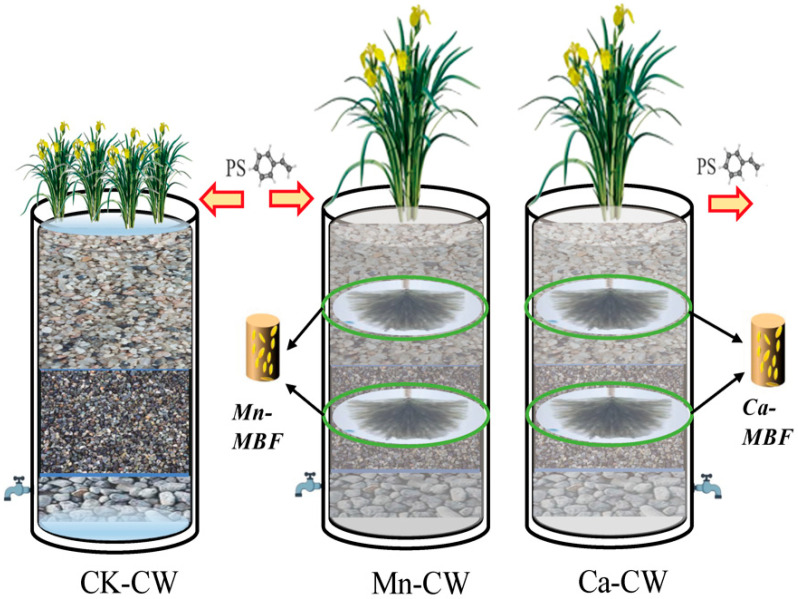
Experimental device schematic. (CK-CW: Synthetic wastewater without MBF bio-nest; Mn-CW: manganese-modified BF (Mn-MBF) in synthetic wastewater; Ca-CW: calcium-modified BF (Ca-MBF) in synthetic wastewater). Reprinted with permission from Ref. [38].

The wetlands operated under an intermittent flow regime with alternating influent and effluent directions. Each cycle involved an inlet volume of 3 L and a hydraulic retention time (HRT) of 72 h. Iris pseudacorus was selected as the wetland plant, with six uniformly grown seedlings planted in each group. The control group (without MBF bio-nests) was designated CK-CW, whereas the experimental groups, enriched with manganese and calcium after contact oxidation tests, were designated Mn-CW and Ca-CW, respectively. A 30-day acclimation period preceded the formal experimental operation, which lasted for 180 days after system stabilization. The schematic diagram of the wetland configuration is presented in Figure 1, and the properties of polystyrene nanoplastics (PS NPs) are provided in the Supporting Information (Figure S3).

The influent was prepared as synthetic wastewater simulating effluent from an A/O-treated domestic sewage system. Target influent water quality parameters were: COD 40–100 mg L^−1^, ammonia nitrogen 5–10 mg L^−1^, and total phosphorus ~2 mg L^−1^, consistent with reported values for treated domestic wastewater [36]. Considering environmental concentrations of micro- and nanoplastics [37], the PS NP concentration was set at 1 mg L^−1^. The theoretical pollutant concentrations in the influent are summarized in Table S2, with the components detailed in Table S3. Analytical methods for water quality assessment are provided in Table S4 and Supplementary Information S1.

### 2.3. The Substrate Enzyme

Given the slow biofilm formation during the early stage of wetland operation, substrate enzyme activity in this experiment was measured starting from the second month of operation, with sampling conducted twice per month. Substrate samples were collected vertically (0–10 cm depth) from the central area of each wetland module using a sterile corer to avoid edge effects. For each treatment (*n* = 3 biological replicates), subsamples from multiple points within a replicate were pooled, sieved (2 mm), and homogenized into a composite sample. This multi-point mixed sampling strategy was employed to mitigate spatial heterogeneity and minimize micro-environmental bias, thereby ensuring a representative analysis of system-wide properties. A range of substrate enzymes was quantified, including dehydrogenase, with organic matter degradation, urease, ammonia monooxygenase, nitrite oxidase, nitrate reductase, and nitrite reductase, with nitrogen removal, as well as neutral phosphatase, associated with phosphorus removal. The specific methodologies employed for determining the activity of each enzyme are detailed in Table S5.

Usually, 15 g of substrate was added to 20 mL of water and mixed in a shaker (200 r min^−1^, 20 min) to obtain a turbid solution. The supernatant was centrifuged at 4000 rpm for 15 min and filtered through a 0.45 μm filter head for S-EPS assay; the precipitated portion was repeated with water, centrifugation, and filtration for LB-EPS and TB-EPS assays. Finally, 15 mL of water was added and thoroughly mixed to prepare a 1 g mL^−1^ biofilm suspension, which served as the enzyme solution.

Dehydrogenase (DHA): One milliliter each of enzyme solution, Tris-HCl, glucose, and TCC solution was combined and incubated in the dark at 37 °C for 24 h. The reaction was terminated by adding sulfuric acid, followed by extraction with toluene and centrifugation. The absorbance was measured at 485 nm within 30 min. Urease (UR): To 0.5 mL of enzyme solution, toluene, PB buffer, urea, and KCl solution were added. The mixture was sonicated at room temperature and centrifuged. The supernatant was collected, diluted with color developer, and incubated for 10 min at 25 °C for color development. Absorbance was then measured at 420 nm. Phosphatase (PST): To 0.5 mL of enzyme solution, toluene was added, followed after 15 min by buffer and substrate. The mixture was then incubated at 37 °C for 1 h. The reaction was terminated by NaOH, centrifuged, diluted, and the absorbance was measured at 400 nm.

Nitrogen Cycle Enzyme Activity Assays (AMO, NOR, NAR, NIR): For each enzyme assay, the enzyme solution was mixed with PB buffer, specific reaction substrates, and electron donors (NAR, NIR) and reacted at a constant temperature of 37 °C for 2–4 h. The enzyme solution was mixed with a KCl leaching solution, sonicated for 15 min, and centrifuged. The supernatant was collected, and a nitrite nitrogen chromogenic agent was added. After 20 min of color development at room temperature, the absorbance was measured at 540 nm. After centrifugation, the supernatant was reacted with a nitrite-specific chromogenic agent for 20 min at room temperature, and absorbance was measured at 540 nm.

### 2.4. Microbiological Analyses

Microbiological samples were collected from the river sand layer (15–25 cm depth) of each wetland unit following the 180-day operational period. Genomic DNA was extracted from the MBF bio-nest samples using a commercial microbial DNA extraction kit (OMEGA Bio-Tek, Norcross, GA, USA). The hypervariable V3–V4 region of the bacterial 16S rRNA gene was subsequently amplified via polymerase chain reaction (PCR) with the universal primers 338F (ACTCCTACGGGAGGCAGCA) and 806R (GGACTACHVGGGTWTCTAAT). Amplification and high-throughput sequencing were conducted by Shanghai Parsonage BioTech Co. (Shanghai, China) on an Illumina MiSeq PE300 platform (Illumina, San Diego, CA, USA). Raw sequences were processed using Vsearch software (Version 2.8.1); quality-filtered sequences were clustered into operational taxonomic units (OTUs) at a 97% similarity threshold.

### 2.5. Statistical Analysis

Experimental data are presented as the mean ± standard error (mg L^−1^). All statistical analyses were performed using SPSS software (Version 30.0.0), while data visualization was conducted using Origin software (Version 2024b). The statistical significance of differences among groups was assessed using a one-way analysis of variance (ANOVA) followed by least significant difference (LSD) post hoc tests.

## 3. Result and Discussion

### 3.1. Characteristic of MBF and Bio-Nests

Energy-dispersive X-ray spectroscopy (EDS) analysis revealed that the primary elemental composition of the unmodified bio-carrier (BF) consisted of O (48.23%), Si (26.56%), Al (7.52%), Fe (5.49%), Ca (4.82%), and Mg (2.87%) (Table S6). The predominance of O and Si indicates that SiO_2_ was the main component of BF, a finding consistent with previous research [39]. The successful coating of Ca^2+^ ions onto the fibers was demonstrated by a marked 10.60% elevation in calcium content in the Ca-MBF sample compared to the initial BF, demonstrating that Ca ions were successfully coated onto fibers. Simultaneously, the Mn element increased significantly from 0% to 6.92%.

EDS analysis and SEM observations confirmed successful BF modification, with increased metal content and improved surface attachment (Figures S4 and S5). Following the culturing period, MBF bio-nests demonstrated significantly greater biomass coverage than the unmodified BF, which remained smooth and exhibited poor biomass retention. Specifically, Ca-MBF bio-nests displayed strong biomass adsorption capabilities and facilitated efficient substance exchange. In contrast, Mn-MBF bio-nests formed cohesive, dense biofilms characterized by robust microbial attachment (Table S7). In actual testing, MBF bio-nests were tightly wrapped with adhesive biofilms, achieving film attachment rates over twice that of BF. Modified carriers (Ca-MBF, Mn-MBF) displayed rough, granular surfaces, enhancing surface area, microbial fixation, and bio-affinity, supported by significantly increased metal content and biofilm formation compared to unmodified BF.

The development of Ca- and Mn-MBFs with elevated surface roughness and strong biofilm adhesion significantly augmented the treatment performance of constructed wetlands. The integration of these bio-nests resulted in a substantial enhancement of pollutant removal, with COD increasing by up to 18.88% and TP by up to 17.94% compared to unmodified systems [38]. Under nano-plastic exposure, untreated wetlands exhibited inhibited nitrification, whereas Mn-MBF promoted the transformation of NO_2_^−^-N and NO_3_^−^-N, maintaining stable NH_4_^+^-N and TN removal rates above 80%. EPS secretion was markedly elevated (62.29–112.11% in Mn-MBF and 17.04–38.32% in Ca-MBF), enhancing wetland biofilm stability. Furthermore, MBF bio-nests strengthened plant antioxidant defenses, reduced membrane lipid peroxidation, and lowered POD, CAT, and MDA levels. Chlorophyll synthesis was also stimulated, improving plant health and resilience. Overall, MBF bio-nests, particularly Mn-MBF, effectively mitigated nano-plastic-induced stress and significantly improved wetland decontamination efficiency.

### 3.2. Effect on Enzyme Activity of Substrate

Constructed enzymes refer to the collective group of enzymes synthesized by microorganisms, secreted by plant roots, and adsorbed onto the substrate surfaces within wetlands. These enzymes serve as indicators of biochemical reactions and microbial metabolic activity within wetland systems [40]. Previous studies have demonstrated that polystyrene nano-plastics (PS NPs) can adversely impact microbial metabolism, enzyme functionality, and plant growth in wetlands, thereby impairing the transformation processes involved in carbon, nitrogen, and phosphorus removal [41].

#### 3.2.1. Ammonia Monooxygenase and Nitrite Oxidoreductase

**Figure 2:**
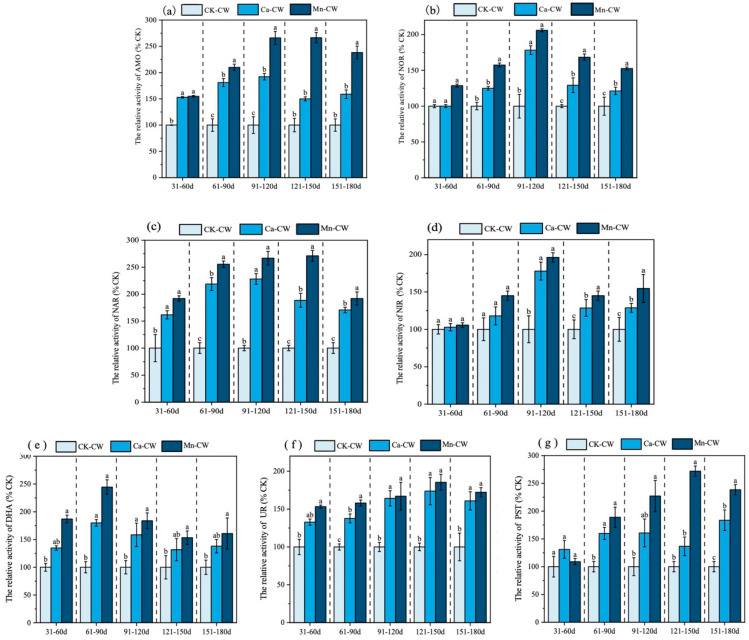
Relative activity of substrate enzymes in CWs exposed to PS NPs. (**a**) AMO, (**b**) NOR, (**c**) NAR, (**d**) NIR, (**e**) DHA, (**f**) UR, and (**g**) PST devices with different labeling letters indicate significant differences (*p* < 0.05).

Ammonia monooxygenase (AMO) catalyzes the oxidation of ammonia nitrogen to hydroxylamine during nitrification [42], while nitrite oxidoreductase (NOR) facilitates the subsequent conversion of nitrite nitrogen to nitrate nitrogen, with its activity strongly correlated to the overall nitrification rate [32]. In this study, the activities of AMO and NOR were quantified across three device groups at different experimental stages (Figure 2).

As shown in Figure 2a, AMO activity in all treatment groups consistently exceeded that of CK-CW, with the highest relative activity observed in Mn-CW. Compared with CK-CW, Mn-CW exhibited increases of 55% (*p* < 0.05), 110% (*p* < 0.05), 166% (*p* < 0.05), 166% (*p* < 0.05), and 138% (*p* < 0.05) across different stages. Similarly, Ca-CW demonstrated respective increases of 53% (*p* < 0.05), 81% (*p* < 0.05), 92% (*p* < 0.05), 50% (*p* < 0.05), and 59% (*p* < 0.05) relative to CK-CW. These results indicate that Mn-MBF bio-nests substantially enhance AMO activity within the constructed wetland system. Elevated AMO activity promotes the initial nitrification step by facilitating dechlorination and dehydrogenation reactions, thereby enabling the efficient transformation of NH_4_^+^-N to hydroxylamine and subsequently improving nitrogen removal efficiency [43]. The findings suggest that MBF bio-nests foster greater microbial abundance and metabolic activity, enhancing system stability and resilience to nanoplastic-induced stress.

As illustrated in Figure 2b, the relative NOR activity in Mn-CW increased by 29% (*p* > 0.05), 58% (*p* < 0.05), 106% (*p* < 0.05), 68% (*p* < 0.05), and 53% (*p* < 0.05) across the respective stages, while Ca-CW exhibited increases of 0.19% (*p* > 0.05), 25% (*p* > 0.05), 78% (*p* < 0.05), 29% (*p* < 0.05), and 21% (*p* < 0.05) compared to CK-CW. These findings indicate that NOR activity was elevated in both treatment groups relative to CK-CW, with a more pronounced enhancement observed in Mn-CW. NOR facilitates the oxidation of NO_2_^−^-N to NO_3_^−^-N, representing the second step of the nitrification process. The markedly higher NOR activity in Mn-CW promoted the continuous conversion of NO_2_^−^-N and indirectly enhanced NH_4_^+^-N transformation, which is consistent with the significantly higher NH_4_^+^-N removal rate observed in this group throughout the experimental cycle [38]. In contrast, NOR activity in Ca-CW exhibited a fluctuating pattern, initially increasing and subsequently decreasing, mirroring the temporal variation of NO_3_^−^-N concentration in the effluent [38]. This inconsistency suggests that the nitrification process in Ca-CW was less efficient than in Mn-CW, highlighting the superior operational stability of Mn-CW from a substrate-driven perspective.

#### 3.2.2. Effect on Nitrite Reductase and Nitrate Reductase Activities

Denitrifying enzymes play a critical role in the denitrification process, a key pathway for nitrogen removal in constructed wetlands. Specifically, nitrate reductase (NAR) catalyzes the reduction of nitrate (NO_3_^−^-N) to nitrite (NO_2_^−^-N) [44], whereas nitrite reductase (NIR) subsequently reduces nitrite to nitric oxide (NO) [45].

As shown in Figure 2c, the relative NAR activity in Mn-CW increased by 92–171% (*p* < 0.05) compared with CK-CW, while Ca-CW exhibited an increase of 62–128% (*p* < 0.05). These results indicate that incorporating MBF bio-nests partially mitigated the inhibitory effects of PS NPs on NAR activity. Both Mn-CW and Ca-CW demonstrated a consistent increase in NAR and NIR activities during 30–120 days of operation, suggesting that MBF bio-nest-augmented wetlands possess inherent resistance to nanoplastic-induced stress, thereby enabling stable denitrification within the system. In the middle to late operational stages (90–180 days), the relative NIR activities of Mn-CW and Ca-CW were significantly higher than those of CK-CW (Figure 2d). Specifically, Mn-CW showed increases of 96% (*p* < 0.05), 45% (*p* < 0.05), and 55% (*p* < 0.05) during the 90–120-, 120–150-, and 150–180-day intervals, respectively, while Ca-CW exhibited increases of 78% (*p* < 0.05), 29% (*p* < 0.05), and 29% (*p* < 0.05) over the same periods.

Previous studies have demonstrated that PS NP exposure in constructed wetlands promotes the generation of excessive reactive oxygen species, leading to structural damage of enzyme proteins and phospholipid membranes [46]. Consequently, prolonged exposure (120–180 days) resulted in a gradual decline in substrate enzyme activities in both Mn-CW and Ca-CW. Overall, the introduction of MBF bio-nests significantly enhanced the activities of denitrifying enzymes (NAR and NIR) in the short term, but sustained nano-plastic stress ultimately diminished these activities. Nevertheless, when considering AMO and NOR collectively, both treatment systems maintained robust resistance to nano-plastic stress, with substrate enzyme activities remaining consistently higher than those observed in conventional constructed wetlands.

#### 3.2.3. Dehydrogenases, Ureases, and Phosphatases

Substrate-associated enzymes play a pivotal role in pollutant removal within constructed wetlands. Among them, phosphatase (PST) facilitates the hydrolysis of phosphate esters, thereby contributing to phosphorus removal [41]. Urease (UR) activity exhibits a strong positive correlation with Kjeldahl nitrogen removal [47], and dehydrogenase (DHA), which participates in the degradation of organic matter, serves as a key indicator of overall biodegradation performance [48].

As shown in Figure 2e, the relative DHA activities in Mn-CW increased by 87% (*p* < 0.05), 145% (*p* < 0.05), 84% (*p* < 0.05), 54% (*p* < 0.05), and 61% (*p* < 0.05), whereas Ca-CW exhibited respective increases of 35% (*p* > 0.05), 80% (*p* < 0.05), 58% (*p* < 0.05), 32% (*p* > 0.05), and 38% (*p* > 0.05) compared with CK-CW. Overall, DHA activity significantly increased (*p* < 0.05) across the testing period in both treatment groups relative to CK-CW, indicating that MBF bio-nest augmentation alleviated the inhibitory effects of PS NPs on DHA activity. The highest enhancement was observed in Mn-CW. However, DHA activity in both Mn-CW and Ca-CW followed a temporal pattern characterized by an initial increase (30–90 days) followed by a decline (90–180 days), suggesting that prolonged nanoplastic exposure gradually suppresses dehydrogenase activity, thereby impairing the wetland system’s capacity for organic matter degradation.

Urease plays a crucial role in the soil nitrogen cycle by catalyzing the hydrolysis of urea into NH_4_^+^-N, thereby facilitating the breakdown of nitrogen-containing organic matter [49]. As shown in Figure 2f, the relative activities of UR increased under nanoparticle (NP) exposure, with the magnitude following the order Mn-CW < Ca-CW < CK-CW. Specifically, UR activity in Mn-CW increased by 53–72% (*p* < 0.05), while Ca-CW exhibited an increase of 33–74% (*p* < 0.05). Toward the end of the experimental period, however, UR activity in both Mn-CW and Ca-CW slightly declined, likely due to the cumulative inhibitory effect of prolonged PS NP exposure. As a key enzyme responsible for converting organic nitrogen to inorganic nitrogen, a reduction in UR activity can impede the denitrification process and consequently diminish nitrogen removal efficiency in wetlands. Consistent with these findings, previous studies have reported a significant decrease in UR activity in constructed wetlands after 60 days of nanoparticle exposure, accompanied by the accumulation of organic nitrogen in the effluent [50].

Phosphatase (PST) promotes the hydrolysis of organic phosphorus and is an important substance involved in biological phosphorus metabolism. As shown in Figure 2g, compared with CK-CW, the relative PST activities in Mn-CW increased by 9% (*p* > 0.05), 89% (*p* < 0.05), 127% (*p* < 0.05), 172% (*p* < 0.05), and 138% (*p* < 0.05) across five experimental phases. In Ca-CW, the increases were 31% (*p* > 0.05), 60% (*p* < 0.05), 61% (*p* < 0.05), 37% (*p* > 0.05), and 84% (*p* < 0.05), respectively. These results demonstrate that PST activity was enhanced in both treatment groups relative to CK-CW, with a more pronounced improvement observed in Mn-CW. During the early experimental stage (30–60 days), differences in PST activity among the groups were not significant, and PST activity displayed a positive correlation with total phosphorus (TP) removal efficiency [34], consistent with the observed TP removal results [51]. In the later stages, PST activity in Mn-CW exhibited a steady and significant increase compared to CK-CW, indicating that Mn-MBF and Ca-MBF bio-nests effectively alleviated PS NP-induced inhibition of PST activity, with Mn-MBF showing a stronger enhancement effect.

Overall, both Ca and Mn bio-nests contributed to improving phosphatase activity, with Mn-CW achieving superior enhancement. This improvement was reflected not only in higher phosphorus conversion efficiency but also in a more stable enzymatic response. Specifically, for nitrogen-transforming enzymes (AMO, NOR, NAR, and NIR), Mn-CW bio-nests maintained a consistent trend of increasing activity during the initial phase (0–120 days), followed by a gradual decrease in the later phase (120–180 days), highlighting their capacity to sustain enzymatic stability under nano-plastic stress.

#### 3.2.4. Correlation of Substrate Enzymes and Ecological Indicators

**Figure 3:**
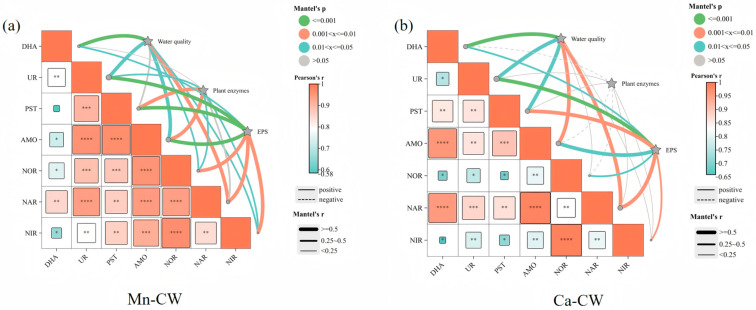
Correlation between substrate enzyme activities and water quality, plant enzymes, and EPS under PS NPs exposure (**a**) Mn-CW (**b**) Ca-CW. * *p* > 0.05, ** 0.01 < *p* < 0.05, *** 0.001 < *p* < 0.01, **** *p* < 0.001.

Based on previous studies, substrate enzyme activities were analyzed alongside water quality parameters, plant characteristics, and extracellular polymeric substance (EPS) content using a Mantel analysis. This analysis was conducted for both Mn-CW and Ca-CW treatment groups, using CK-CW as the control. Figure 3 illustrates the correlations between substrate enzyme activities and various characteristics of the constructed wetland systems. In constructed wetlands, water quality indicators and substrate enzyme activities form a dynamic, interactive “drive–feedback” mechanism: enzymes catalyze pollutant transformations that directly improve water quality, while shifts in water quality, in turn, regulate enzymatic activity [52]. The average COD and TN removal rates in Mn-CW and Ca-CW were improved by 14.15–18.88% and 13–19%, respectively, compared to the control group (CK-CW), while high TP removal efficiency was also maintained, achieving optimal phosphorus elimination. Furthermore, both systems significantly enhanced the transformation of NO_2_^−^-N, NO^3−^-N, and NH_4_^+^-N, sustaining efficient coupled nitrification–denitrification processes [38].

Previous research has shown that polyethylene microplastics can adsorb dehydrogenase, impeding enzyme–substrate interactions and leading to decreases in COD removal efficiency and methane yields by 17.4–30.4% and 17.2–28.4% [53]. The Mantel analysis in this study revealed a strong correlation (*p* < 0.001) between dehydrogenase (DHA) activity and water quality parameters in both Mn-CW and Ca-CW treatments. As DHA is involved in the degradation of organic matter, its strong association with improved COD removal aligns with its role in evaluating organic pollutant breakdown. Additionally, nitrate reductase (NAR) exhibited a significant correlation (0.001 < *p* < 0.01), consistent with its catalytic function in reducing nitrate (NO^3−^-N) to nitrite (NO_2_^−^-N), achieving a near-complete conversion efficiency (~100%). Overall, water quality indicators in both treatment groups were more strongly correlated with nitrogen-transforming enzymes, highlighting that the bio-nest structures of Mn-CW and Ca-CW play a crucial role in sustaining enzyme activity and supporting the long-term operational stability of wetland water purification processes.

Plant root secretions directly stimulate the synthesis of plant-derived enzymes [38]. Specifically, the exudation of organic acids (e.g., oxalic acid, citric acid), sugars, and phenolic compounds from roots can enhance microbial secretion of hydrolytic enzymes (e.g., phosphatases, ureases) as well as oxidoreductases (e.g., dehydrogenases, peroxidases) [38]. Interestingly, in the Mn-CW treatment group, plant enzymes and substrate enzymes formed a relatively integrated functional network. Most substrate enzymes exhibited significant correlations with plant enzyme activities. Notably, phosphatase (PST), which catalyzes the hydrolysis of phosphate esters, and ammonia monooxygenase (AMO), which facilitates the conversion of ammonia nitrogen to hydroxylamine during nitrification, both displayed positive correlations with plant enzymes (0.001 < *p* < 0.01). In contrast, the Ca-CW treatment group not only lacked positive correlations but also exhibited partial negative correlations between plant and substrate enzyme activities. The incorporation of bio-nests effectively mitigated nanoplastic-induced stress in both Mn-CW and Ca-CW systems. This mitigation was reflected in stabilized plant photosynthetic activity, evidenced by a significant reduction in malondialdehyde (MDA) content, which indicates alleviation of membrane lipid peroxidation. Furthermore, bio-nest addition enhanced chlorophyll biosynthesis, resulting in higher levels of chlorophyll a, chlorophyll b, and total chlorophyll compared with CK-CW [38].

Substrate-associated enzymes (e.g., urease, phosphatase, dehydrogenase) are primarily secreted by microorganisms and constitute the core functional components of extracellular polymeric substances (EPS) [38]. Microorganisms form biofilms by secreting EPS, within which extracellular enzymes are immobilized in a polymeric substrate, preventing enzymatic loss and preserving activity stability [38]. The incorporation of MBF bio-nests significantly enhanced EPS secretion. During the 0–180-day operational cycle, the total EPS content in Mn-CW and Ca-CW was markedly higher than that in CK-CW (*p* < 0.05), with increases of 62.29–112.11% in Mn-CW and 17.04–38.32% in Ca-CW, respectively. The enhancement effect was more pronounced in the Mn-MBF system [38]. Moreover, the polysaccharide–protein network within EPS served as a protective buffer against environmental stresses (e.g., pH fluctuations, heavy metals), thereby maintaining the conformational stability and functional integrity of substrate enzymes. Correlation analysis revealed strong associations (*p* < 0.001) between EPS secretion and the activities of AMO, PST, and UR, as well as moderate correlations (0.001 < *p* < 0.01) with NOR, NAR, and NIR in the Mn-treated group. The porous structure of EPS facilitated pollutant adsorption, enhanced enzyme–substrate binding efficiency, and improved catalytic performance [38]. In contrast, the correlations observed in the Ca-treated group were weaker than those in the Mn-treated group. Urease (UR) activity demonstrated a significant positive correlation with nitrogen removal, suggesting that manganese modification induced microorganisms to secrete greater amounts of polysaccharides (PS) and proteins (PN), thereby reinforcing the structural stability and functionality of the wetland biofilm.

### 3.3. Microbial Diversity Analysis

#### 3.3.1. Analysis of Community Diversity and Species Difference

The Chao1 index is commonly employed as an indicator of habitat richness, whereas the Shannon and Simpson indices are reflective of overall microbial community diversity. As presented in Table 1, the incorporation of MBF bio-nests markedly enhanced both microbial richness and diversity within the constructed wetland systems. Specifically, Chao1 values increased by 32.69% in Mn-CW and 1.04% in Ca-CW, while the Observed Species index rose by 32.72% and 1.13%, respectively, relative to CK-CW. Similarly, Shannon diversity increased by 8.60% in Mn-CW and 3.45% in Ca-CW. Collectively, these results indicate that the deployment of MBF bio-nests can significantly promote microbial community diversity and richness. Importantly, however, none of the four diversity metrics in Ca-CW exhibited statistically significant improvements compared with the control group, underscoring that Mn-MBF bio-nests exerted a more pronounced and beneficial influence on microbial community structure.

**Figure 4:**
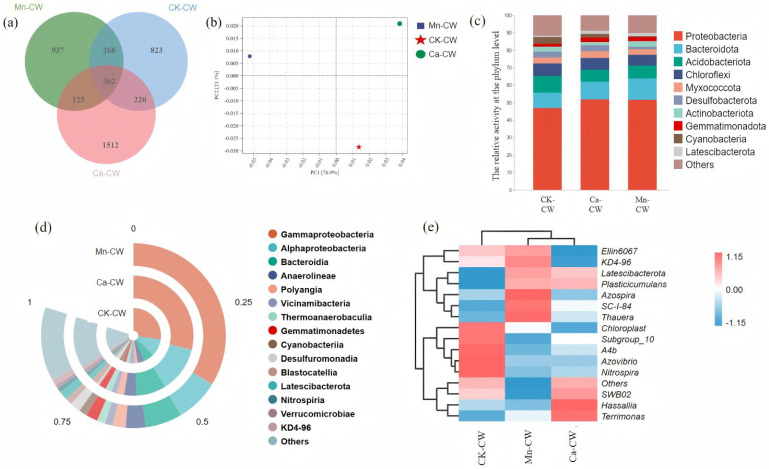
Microbial communities of CWs exposed to PS NPs (**a**) Wayne plots, (**b**) PCA analysis of species differences, (**c**) top 10 phylum-level species in relative abundance, (**d**) top 15 phylum-level species in terms of degree, and (**e**) top 15 genus-level species in terms of degree.

The Venn diagram provides a visual representation of differences in microbial community composition among the wetland groups (Figure 4a). A total of 4247 OTUs were identified across all samples, with only 362 OTUs (8.52%) shared among the three groups, indicating substantial variation in microbial community structures. Additionally, Ca-CW and CK-CW shared 268 OTUs (6.31% of the total), while Mn-CW and CK-CW shared 220 OTUs (5.18%), suggesting that both Mn-MBF and Ca-MBF bio-nests altered microbial structural composition, with the Mn-MBF treatment inducing more pronounced changes. Principal Component Analysis (PCA) was employed to assess the differences and relative distances in microbial composition between samples. In PCA plots, closer sample clustering indicates higher similarity in microbial community structure. As shown in Figure 4b, CK-CW was positioned farther from both Mn-CW and Ca-CW, confirming that MBF bio-nest incorporation significantly altered microbial community composition. Furthermore, the greater separation between Mn-CW and Ca-CW suggests that the two modification strategies exerted distinct influences on microbial structural composition.

#### 3.3.2. The Structure of Microbial Community

The microbial community structure at the phylum level is presented in Figure 4c. Proteobacteria exhibited the highest relative abundance across all wetland systems, accounting for 46.80% in CK-CW, 51.42% in Mn-CW, and 51.80% in Ca-CW. This was followed by Bacteroidota (8.76–12.31%), Acidobacteriota (6.93–9.63%), Chloroflexi (6.41–7.24%), Myxococcota (3.09–3.81%), and Desulfobacterota (1.52–3.62%). Proteobacteria are known to play a central role in nitrogen and phosphorus removal as well as organic matter degradation [54], with higher proportions observed in Mn-CW and Ca-CW compared to CK-CW, supporting the enhanced pollutant removal performance of MBF bio-nest-augmented wetlands. Bacteroidota, which accounted for a higher percentage in Mn-CW, are capable of secreting large amounts of proteins (PN) that facilitate microbial aggregation and biofilm formation. This increase in Bacteroidota abundance corresponds with the elevated PN content observed in EPS fraction analyses [38]. Additionally, Bacteroidota include bacterial taxa involved in nitrogen and phosphorus removal, closely linked to the denitrification process [55]. The enrichment of Bacteroidota in both Mn-CW and Ca-CW suggests that MBF bio-nest addition promotes conditions favorable for denitrification. Moreover, Acidobacteriota [56] and Chloroflexi [57] have been identified as typical denitrifying microorganisms capable of thriving under harsh environmental conditions. The selective enrichment of denitrifying Bacteroidota in Mn-CW likely underpins the high NO_3_^−^-N removal efficiency observed during the later operational stages of the wetland systems [38].

The microbial community structure at the phylum level is shown in Figure 4d. The dominant phyla were Gammaproteobacteria (γ-amoeba), Alphaproteobacteria (α-amoeba), Bacteroidia (Bacteroidia), Anaerolineae (Anaerobic cordyceps), and Polyangia (Polyangia), with corresponding relative abundances of 34.10–42.26%, 9.54–15.36%, 7.61–9.98%, 4.22–4.95% and 2.26–2.85%, respectively. There were differences in the percentage of each dominant phylum in the wetland, in which the percentage of Gammaproteobacteria and Alphaproteobacteria anamorphic phyla in Mn-CW and Ca-CW was significantly higher than that in CK-CW, and it was the highest in the Ca-CW group. Alphaproteobacteria contain more nitrogen-fixing bacteria and phosphorus-aggregating bacteria, which are conducive to the removal of nitrogen and phosphorus in the wetland, among which Alphaproteobacteria are autotrophic nitrifying bacteria, which play an important role in nitrification, and Gammaproteobacteria are parthenogenetic heterotrophic bacteria, which are the main participants in COD degradation [58]. Gammaproteobacteria gave the wetland system a better water purification ability. Meanwhile, Anaerolineae has a denitrification function [59], and Mn-CW and Ca-CW contained a high percentage of Anaerolineae, which explains their high nitrate nitrogen removal rate. In addition, the relative abundance of Bacteroidia also increased with the addition of MBF bio-nests, and the abundance of Bacteroidia in Mn-CW and Ca-CW increased by 31.14% and 5.91%, respectively, compared with CK-CW, and the higher Bacteroidia occupancy ratio was beneficial for the denitrification process and the removal of difficult-to-degrade organic matter [55].

The relative abundance of the top 15 genera is presented in Figure 4e. The three most dominant genera were hydrolytic bacteria (SC-I-84), *Thauera*, and *Plasticicumulans*, with relative abundances of 3.88–7.20%, 2.91–8.63%, and 3.09–3.54%, respectively. SC-I-84 belongs to the phylum Ascomycota [60], while *Thauera* and *Plasticicumulans* are recognized for their roles in denitrification and for promoting EPS secretion [61]. The coexistence of Ascomycota species and aerobic denitrifying bacteria ensures effective pollutant removal and stable operational performance of constructed wetlands. Notably, the microbial communities of the novel artificial wetlands (Mn-CW and Ca-CW) differed markedly from those of the conventional wetland (CK-CW) at the genus level, indicating that the addition of MBF bio-nests altered microbial community structure. Among these, Mn-CW exhibited the highest relative abundances of SC-I-84, *Thauera*, and *Plasticicumulans*. Specifically, *Thauera* abundance in Mn-CW exceeded that in CK-CW and Ca-CW by 5.72% and 4.05%, respectively. *Thauera* has been reported to play a crucial role in nitrogen pollutant removal and in degrading recalcitrant compounds [62], suggesting that its enrichment in Mn-CW may contribute to the system’s superior denitrification performance.

The abundance of *SC-I-84* in CK-CW, Mn-CW, and Ca-CW was 3.88%, 7.20%, and 5.38%, respectively, with Mn-CW containing approximately twice the abundance found in CK-CW. *SC-I-84* is known for its nitrogen fixation capability, environmental adaptability, disease resistance, and strong tolerance to stress [63]. The elevated abundance of *SC-I-84* in Mn-CW likely enhances the system’s resilience to nanoplastic-induced stress, thereby supporting more robust denitrification and overall wetland stability.

### 3.4. Functional Microbial Analysis

**Figure 5:**
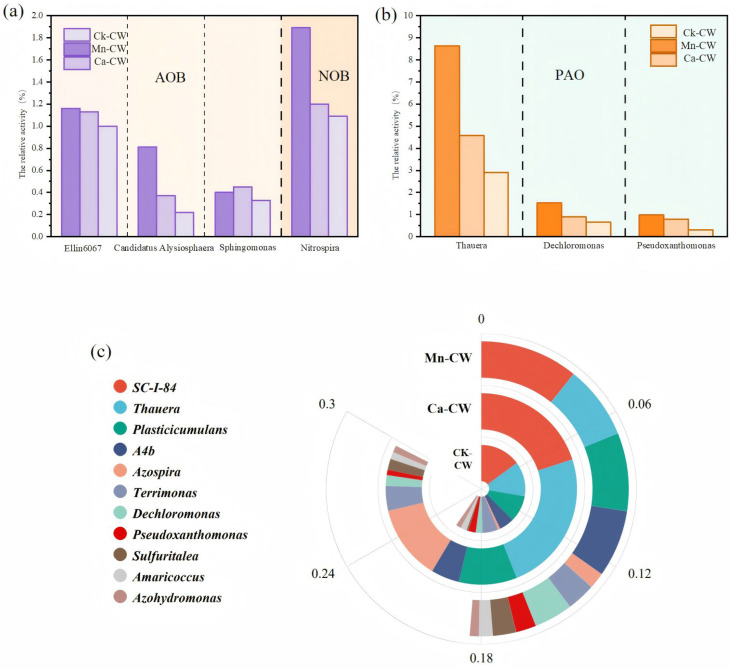
Histogram of relative abundance of nitrifying functional bacteria and polyphosphate bacteria (**a**) nitrifying bacteria, (**b**) polyphosphate bacteria, and (**c**) differences in the distribution of denitrifying functional bacteria in each group.

In this study, the microbial abundances of key nitrogen- and phosphorus-removing bacteria were quantified, including polyphosphate-accumulating organisms (PAOs), denitrifying bacteria (DNBs), nitrite-oxidizing bacteria (NOBs), and ammonia-oxidizing bacteria (AOBs). The distribution and relative abundances of these functional bacterial groups are presented in Figure 5 and Table 2.

As shown in Figure 5a, AOBs detected in the substrate samples from the three wetland systems included Ellin6067 [64], heterotrophic denitrifiers such as *Candidatus Alysiosphaera* [65], and *Sphingomonas* [66], while NOBs were primarily represented by *Helicobacter nitrificus* and *Nitrospira*. The total abundance of nitrifying bacteria was 1.55%, 2.37%, and 1.95% in CK-CW, Mn-CW, and Ca-CW, respectively. These results indicate that MBF bio-nest incorporation promoted the growth of nitrifying functional bacteria, enhancing NH_4_^+^-N removal efficiency by modulating microbial community structure and thereby facilitating the nitrification process. This finding is consistent with the highest NH_4_^+^-N removal rates observed in Mn-CW and Ca-CW throughout the operational cycle [38]. Among the AOBs, *Candidatus Alysiosphaera* and *Sphingomonas* were typical heterotrophic ammonia oxidizers present at relatively low abundances, whereas Ellin6067, an autotrophic AOB, was more dominant across all wetlands, contributing significantly to NH_4_^+^-N removal, with the highest relative abundance (1.16%) detected in Mn-CW. The relative abundance of Nitrospira (NOB) followed the order Mn-CW (1.89%) > Ca-CW (1.20%) > CK-CW (1.09%). The increased abundance of Nitrospira in MBF bio-nest-enhanced wetlands compared to the control system indicates more active nitrite oxidation. This enrichment of NOBs is likely a key factor underlying the observed differences in nitrification performance among the treatment groups.

As shown in Figure 5b, the predominant phosphorus-removing bacteria identified in the three wetland groups included *Thauera* [67], *Dechloromonas* spp. [68], and *Pseudoxanthomonas* spp. [69]. *Thauera* is recognized as both a typical denitrifying bacterium and a phosphorus-accumulating organism [67]. In this study, the relative abundance of *Thauera* varied significantly among the systems, with the highest observed in Mn-CW (8.62%), followed by Ca-CW (4.59%) and CK-CW (2.91%). The other two phosphorus-accumulating genera, *Dechloromonas* and *Pseudoxanthomonas*, also exhibited higher relative abundances in Mn-CW and Ca-CW compared to CK-CW. *Pseudoxanthomonas*, known for its ability to remove phosphorus under anoxic conditions [70], was most abundant in Mn-CW (0.98%), approximately three times higher than in CK-CW (0.32%). Similarly, the relative abundance of *Dechloromonas* increased in the bio-nest-amended systems, with values of 0.65%, 1.53%, and 0.89% for CK-CW, Mn-CW, and Ca-CW, respectively. These findings indicate that both Mn-MBF and Ca-MBF bio-nests positively influenced the enrichment of phosphorus-removing bacteria. When combined with phosphorus removal performance data, the enhanced TP removal efficiency observed in Mn-CW and Ca-CW relative to CK-CW can be attributed to the elevated abundances of polyphosphate-accumulating organisms, particularly *Thauera*, *Dechloromonas*, and *Pseudoxanthomonas*. The superior phosphorus removal observed in Mn-CW compared to Ca-CW is primarily linked to the greater enrichment of these key phosphorus-removing taxa.

As shown in Figure 5c, a total of 11 functional denitrifying bacterial genera were detected across the wetland systems. Among them, *Hydrophilus (SC-I-84)*, *Thauera*, *Plasticicumulans* [71], *Azospira* [72], *A4b*, *Hygrophilus (Terrimonas)*, and *Dechloromonas* were the predominant taxa, while *Sewerococcus (Amaricoccus)*, nitrogen-fixing hydrogen-autotrophic Aeromonas (*Azohydromonas*), *Pseudoxanthomonas*, and *Sulfuritalea* [73] were present in lower abundances.

According to Table 3, the total abundance of denitrifying bacteria was 15.45% in CK-CW, 32.83% in Mn-CW, and 21.38% in Ca-CW. These results demonstrate that MBF bio-nest-enhanced wetlands (Mn-CW and Ca-CW) supported a greater enrichment of denitrifying bacteria than the conventional wetland system (CK-CW), with Mn-CW showing the highest enrichment. The increased abundance of denitrifying bacteria in bio-nest systems likely facilitated more efficient denitrification, explaining the nearly complete nitrate nitrogen removal observed in the later operational stages of Mn-CW and Ca-CW.

At the genus level, *Thauera* exhibited the highest relative abundance in Mn-CW (8.63%), significantly exceeding that in Ca-CW (4.59%) and CK-CW (2.91%). Similarly, SC-I-84 was more abundant in Mn-CW (7.20%) and Ca-CW (5.38%) than in CK-CW (3.88%), suggesting that *Thauera* and *SC-I-84* are key contributors to denitrification in bio-nest-modified wetlands. Furthermore, the relative abundance of *Azospira* was markedly higher in Mn-CW (4.58%) compared to CK-CW (0.64%) and Ca-CW (0.32%), indicating that Mn-MBF bio-nests promoted its enrichment. Collectively, these findings suggest that MBF bio-nest incorporation substantially reshaped the community structure of denitrifying bacteria, thereby enhancing denitrification performance in the wetland systems.

### 3.5. Functional Gene Prediction

**Figure 6:**
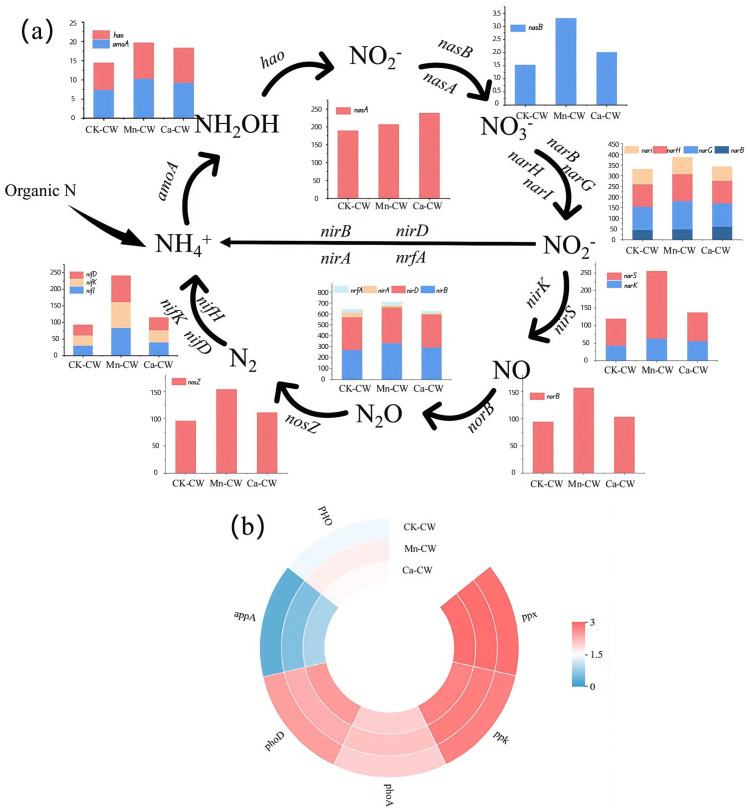
Functional gene abundances predicted based on the PICRUSt2 algorithm. (**a**) Nitrogen cycle-related pathways and nitrogen removal functional genes; (**b**) Functional genes for phosphorus removal.

The incorporation of MBF bio-nests altered the functional microbial composition of the wetland system. To further elucidate these effects, functional gene prediction was performed using the PICRUSt2 algorithm in conjunction with the KEGG database to identify genes associated with denitrification and phosphorus removal. The predicted functional gene profiles are presented in Figure 6.

Nitrogen cycling pathways encompass nitrogen fixation, nitrification, denitrification, dissimilatory nitrate reduction, ammonification, and assimilation. Nitrogen fixation involves the microbial conversion of atmospheric N_2_ into bioavailable NH_4_^+^, primarily mediated by the functional genes nifH, nifK, and nifD. As illustrated in Figure 6a, the abundances of these genes increased following the incorporation of MBF bio-nests, with a particularly pronounced enrichment observed in the Mn-CW treatment. The significantly higher levels of nifH, nifK, and nifD in Mn-CW compared to the other groups indicate that Mn-MBF bio-nests more effectively enhance nitrogen fixation potential within the wetland system.

The nitrification process primarily involves the sequential conversion of NH_4_^+^ to NH_2_OH, NH_2_OH to NO_2_^−^, and NO_2_^−^ to NO_3_^−^, catalyzed by enzymes encoded by amoA, hao, nasA, and nasB. As shown in Figure 6a, the abundances of amoA and hao were elevated in Mn-CW and Ca-CW compared with CK-CW, suggesting that the addition of MBF bio-nests enhanced ammonia oxidation and NH_2_OH oxidation. The nasB gene abundance in Mn-CW increased by 116.20% and 64.00% compared with CK-CW and Ca-CW, respectively, indicating that Mn-MBF bio-nests may specifically promote nasB expression. Conversely, nasA abundance in Ca-CW increased by 25.92% compared with CK-CW and by 15.19% compared with Mn-CW, implying that Ca-MBF bio-nests might favor the expression of the nasA gene. Overall, the combined abundances of nasA and nasB in Mn-CW and Ca-CW were higher than in CK-CW, indicating that MBF bio-nests facilitated the oxidation of nitrosative nitrogen to nitrate, thereby promoting nitrification.

The denitrification process consists of four sequential stages: reduction of NO_3_^−^ to NO_2_^−^, NO_2_^−^ to NO, NO to N_2_O, and finally N_2_O to N_2_. The initial NO_3_^−^ reduction is mediated by the genes narB, narG, narH, and narI. As depicted in Figure 6a, the combined abundances of these genes were higher in Mn-CW and Ca-CW than in CK-CW, with Mn-CW exhibiting the greatest enrichment, indicating that both Mn-MBF and Ca-MBF bio-nests enhanced the first step of denitrification. The subsequent reduction of NO_2_^−^ to NO is encoded by nirS and nirK. The MBF bio-nest addition increased the abundances of both genes, suggesting that bio-nests facilitated this critical reduction step. The final stages of denitrification—NO reduction to N_2_O and N_2_O conversion to N_2_—are encoded by norB and nosZ, respectively. Both gene abundances were notably increased in Mn-CW, demonstrating that Mn-MBF bio-nests were more effective than Ca-MBF bio-nests in promoting NO and N_2_O reduction. Additionally, dissimilatory nitrate reduction, a key step in anaerobic ammonia oxidation-driven denitrification, involves the functional genes nirB, nirD, nirA, and nrfA. Although the overall abundances of these genes did not differ significantly among the three systems, Mn-CW exhibited slightly higher levels of all four genes compared to Ca-CW and CK-CW, suggesting that Mn-MBF bio-nests provided marginally greater support for heterogeneous nitrate reduction.

In this study, six key functional genes associated with phosphorus removal (*ppx*, *ppk*, *phoA*, *phoD*, *appA*, and *PHO*) were selected to evaluate the impact of MBF bio-nest addition on phosphorus removal performance in wetland systems under PS NP exposure (Figure 6b). Among these, *phoA* and *appA* exhibited higher abundances in MBF bio-nest-enhanced wetlands (Mn-CW and Ca-CW), whereas *phoD* was more prevalent in the conventional wetland (CK-CW). The abundances of the remaining phosphorus removal genes (*ppx*, *ppk*, and *phoA*) were relatively similar across CK-CW samples. When considering the total abundance of phosphorus removal functional genes, Mn-CW demonstrated the highest levels, followed by Ca-CW, with CK-CW showing the lowest. These findings suggest that incorporating MBF bio-nests enhances the abundance of phosphorus removal-related genes, thereby potentially improving biological phosphorus removal efficiency, with Mn-MBF bio-nests exhibiting a stronger promoting effect than Ca-MBF bio-nests.

In summary, the incorporation of MBF bio-nests significantly enhanced the abundance of functional genes associated with the nitrogen cycle, thereby promoting key processes including nitrogen fixation, nitrification, denitrification, and heterogeneous nitrate reduction within wetland systems. Notably, Mn-MBF bio-nests exhibited a more pronounced effect, with substantially higher abundances of critical nitrogen cycle genes compared to those observed in conventional constructed wetlands.

## 4. Conclusions

Under continuous NP exposure, MBF bio-nests substantially mitigated ecological stress in constructed wetlands by enhancing substrate enzyme activities, promoting biofilm formation, and restructuring microbial communities. Mn-MBF bio-nests demonstrated stronger functional resilience than Ca-MBF, significantly enriching nitrifying, denitrifying, and phosphorus-removing bacteria, as well as key nitrogen cycle genes. These functional enhancements led to marked improvements in COD, nitrogen, and phosphorus removal performance. Overall, MBF bio-nests provide an effective, scalable substrate modification approach to protect CW systems against emerging nano-plastic contaminants, ensuring long-term pollutant removal efficiency and ecological stability.

## Figures and Tables

**Table 1:** Alpha diversity analysis index.

Groups	Observed Species	Chao1	Shannon	Simpson	Goods Coverage
CK-CW	1673.1	1675.72	9.07418	0.993141	0.999058
Mn-CW	2220.6	2223.59	9.85513	0.996969	0.999464
Ca-CW	1692.1	1693.18	9.38836	0.996123	0.999291

**Table 2:** Relative abundance of nitrifying functional bacteria and phosphate-accumulating bacteria (%).

Functional Type	Genus of Microorganisms	CK-CW	Mn-CW	Ca-CW
AOB	*Ellin6067*	1.00	1.16	1.13
	*Candidatus Alysiosphaera*	0.22	0.81	0.37
	*Sphingomonas*	0.33	0.40	0.45
NOB	*Nitrospira*	1.09	1.89	1.20
PAO	*Thauera*	2.91	8.62	4.59
	*Dechloromonas*	0.65	1.53	0.89
	*Pseudoxanthomonas*	0.32	0.98	0.80

**Table 3:** Relative abundance of denitrifying functional bacteria in groups (%).

Function Types	Microorganisms Genus	CK-CW	Mn-CW	Ca-CW
DNB	SC-I-84	3.88	7.20	5.38
	Thauera	2.91	8.63	4.59
	Plasticicumulans	3.09	3.54	3.52
	Azospira	0.64	4.58	0.32
	A4b	1.73	2.66	1.98
	Terrimonas	1.14	1.52	2.08
	Dechloromonas	0.65	1.53	0.89
	Amaricoccus	0.43	0.83	0.54
	Azohydromonas	0.42	0.69	0.36
	Pseudoxanthomonas	0.32	0.98	0.80
	Sulfuritalea	0.24	0.67	0.92

## Data Availability

Data Availability Statements are available. Data are contained within the article and Supplementary Materials. Figure 1 was reprinted with permission from Ref. [38].

## Supplementary Materials

The following supporting information can be downloaded at: https://www.mdpi.com/article/10.3390/toxics13090800/s1, Figure S1: Schematic diagram of basalt fiber modification process. Figure S2: Schematic diagram of biological nest culture. Figure S3: Constructed wetland Site operation diagrams for the (a) start-up period of the constructed wetland, and (b) the stable operation period of the constructed wetland. Figure S4: The characteristics of PS NPs dispersions solution (a) Potential distribution; (b) Particle size distribution; (c) Fourier transform infrared spectra (FTIR) of PS-NPs dispersion. Figure S5: Schematic diagram of biological nest culture (A) SEM of MBF, (B) SEM of MBF bio-nest, (C) Image of MBF bio-nest. Table S1: Theoretical concentration of pollutant in culture stage of bio-nest. Table S2: Theoretical concentration of pollutants in constructed wetland. Table S3: Components of synthetic wastewater from constructed wetland. Table S4: Test methods of water quality parameters. Table S5: Matrix enzyme activity assays. Table S6: Elemental composition of BF and MBF (%). Table S7: Comparison of membrane hanging effect of BF and MBF. Supplementary Information S1: Detection methods of water quality parameters.

## Nomenclature

NPs	Nano-Plastics
CWs	Constructed Wetlands
MBF	Modified Basalt Fiber
PS	Polystyrene
Ca	Calcium
Mn	Manganese
AMO	Ammonia monooxygenase
NOR	Nitrite oxidoreductase
NAR	Nitrate reductase
NIR	Nitrite reductase
PST	Phosphatase
UR	Urease
DHA	Dehydrogenase
