# Supplementary material for: Constructed Wetlands with Novel Substrate Exposed to Nano-Plastics: Mitigating the Effects of Substrate Enzyme and Ecological Processes
Source: Toxics. 2025 Sep 20;13(9):800. doi: 10.3390/toxics13090800 (PMC12473994; doi:10.3390/toxics13090800)
Supplement: Supplementary file 1 [file toxics-13-00800-s001.zip › toxics-3868593-supplementary.pdf]

Supporting Information

FIGURE S1 SCHEMATIC DIAGRAM OF BASALT FIBER MODIFICATION PROCESS......................2

FIGURE S2 SCHEMATIC DIAGRAM OF BIOLOGICAL NEST CULTURE...3

FIGURE S3 CONSTRUCTED WETLAND SITE OPERATION DIAGRAMS FOR THE (A) START-UP PERIOD OF THE CONSTRUCTED WETLAND, AND (B) THE STABLE OPERATION PERIOD OF THE CONSTRUCTED WETLAND..4

FIGURE S4 THE CHARACTERISTICS OF PS NPS DISPERSIONS SOLUTION (A)POTENTIAL DISTRIBUTION; (B) PARTICLE SIZE DISTRIBUTION; (C) FOURIER TRANSFORM INFRARED SPECTRA (FTIR) OF PS-NPS DISPERSION..5

FIGURE S5 SCHEMATIC DIAGRAM OF BIOLOGICAL NEST CULTURE (A) SEM OF MBF, (B) SEM OF MBF BIO-NEST, (C) IMAGE OF MBF BIO-NEST..6

TABLE S1 THEORETICAL CONCENTRATION OF POLLUTANT IN THE CULTURE STAGE OF BIO-NEST..7

*TABLE S2 THEORETICAL CONCENTRATION OF POLLUTANTS IN CONSTRUCTED WETLAND*7

TABLE S4 TEST METHODS OF WATER QUALITY PARAMETERS...8

TABLE S5 MATRIX ENZYME ACTIVITY ASSAYS..9

TABLE S6 - ELEMENTAL COMPOSITION OF BF AND MBF (%)...9

TABLE S7- COMPARISON OF MEMBRANE HANGING EFFECT OF BF AND MBF.....................10

SUPPLEMENTARY *INFORMATION S1 DETECTION METHODS OF WATER QUALITY PARAMETERS*..11

Figure S1 Schematic diagram of basalt fiber modification process

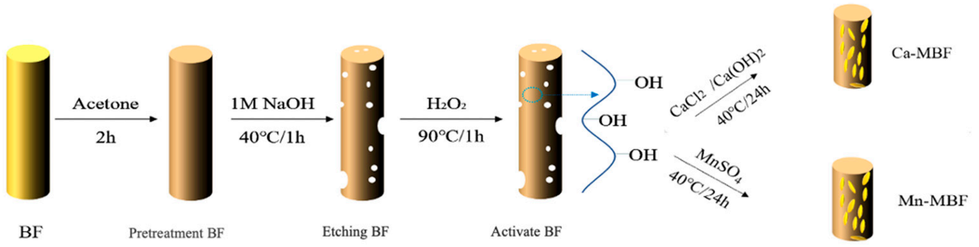

(1) Calcium modification: A 1 M $CaCl_2$ solution and a saturated $Ca(OH)_2$ solution were prepared, then mixed uniformly to obtain a mixture. Subsequently, the PBF was immersed in the mixture and heated in a water bath (40°C, 24 h). Finally, washing and drying PBF with deionized water to obtain Calcium-Modified Basalt Fiber (Ca-MBF).

(2) Manganese modification: 1 M solution of $MnSO_4$ and $KMnO_4$ was mixed homogeneously with a volume ratio of 3:2. Subsequently, the PBF was immersed in the mixture and heated in a water bath (40°C, 24 h). Finally, the resulting PBF was washed with deionized water and dried to obtain Mn-Modified Basalt Fiber (Mn-MBF).

Figure S2 Schematic diagram of biological nest culture

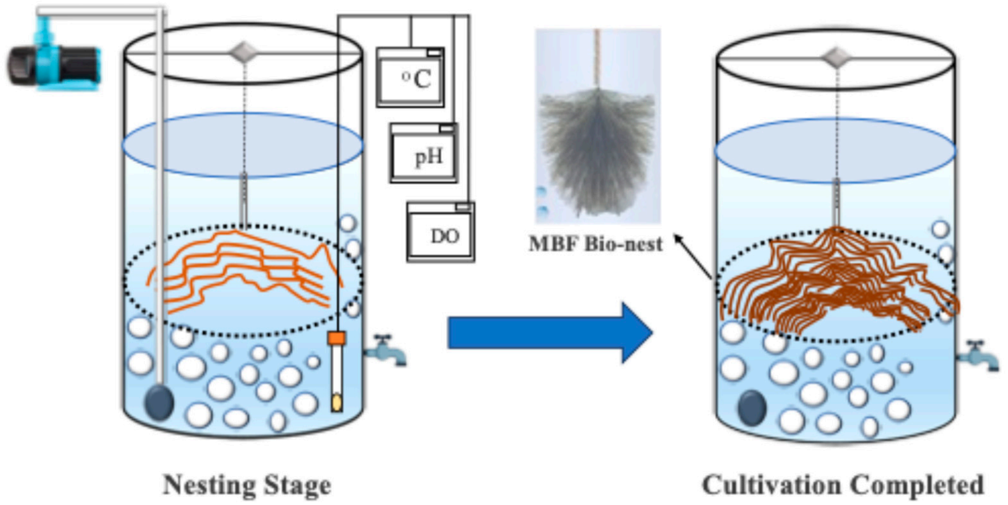

Figure S3 Constructed wetland site operation diagrams for the (a) start-up period of the constructed wetland, and (b) the stable operation period of the constructed wetland

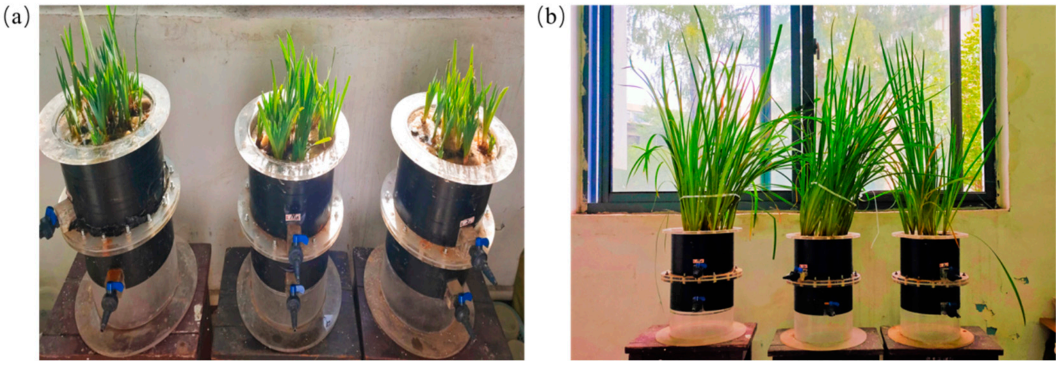

Figure S4 The characteristics of PS NPs dispersion solution: (a) Potential distribution; (b) Particle size distribution; (c) Fourier transform infrared spectra (FTIR) of PS-NPs dispersion

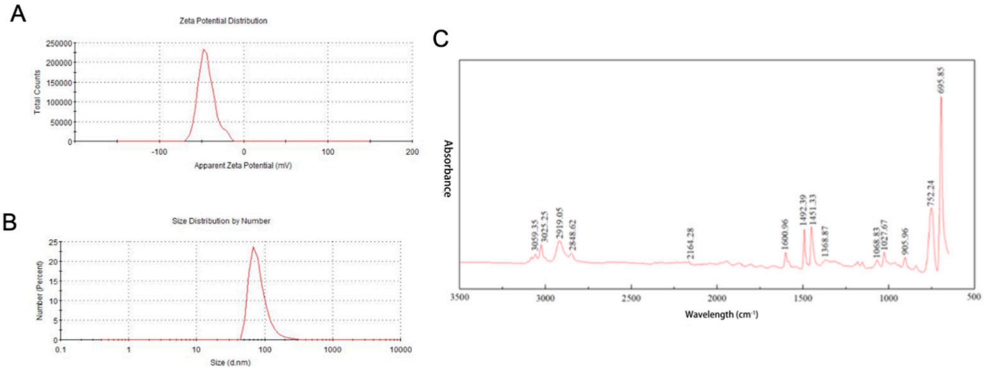

The dispersion of were provided by TianJing Besler Chromatography Technology Development Centre, China, with thickness of 100 nm and dispersion of 250 mg/L. The potential and particle size distributions of the PS NPs were determined by dynamic light scattering (DLS), as shown in Figure S2 and S3, which meets the experimental requirements. The chemical composition of PS NPs was also analyzed using Fourier transform infrared spectroscopy (FTIR) as shown in Figure S4: The peaks of the samples at 695.85 cm^{-1} and 752.24 cm^{-1} corresponded to the C-H out-of-plane deformation vibration on the benzene ring; the characteristic spectral bands at 1451.33 cm^{-1} and 1492.39 cm^{-1} corresponded to the skeletal vibration of the benzene ring; the peaks at 3025.25 cm^{-1} and 3059.35 cm^{-1} corresponded to the stretching vibration of the C-H bond of the benzene ring; the peaks at 2848.62 cm^{-1} and 2919.05 cm^{-1} correspond to the C-H bond stretching vibration peaks in the methylene group. These peaks are consistent with the structure of polystyrene.

Figure S5 Schematic diagram of biological nest culture: (A) SEM of MBF, (B) SEM of MBF bio-nest, and (C) image of MBF bio-nest

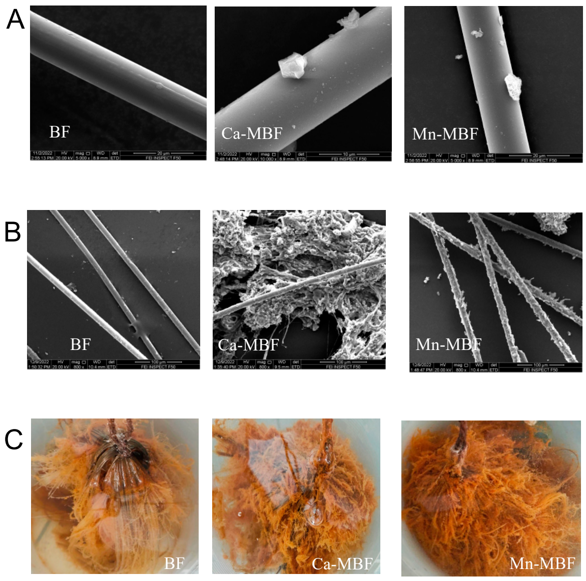

Table S1 Theoretical concentration of pollutant in culture stage of bio-nest

Water quality indicators	COD（mg/L）	TN（mg/L）	NH_4^+-N（mg/L）	TP（mg/L）
Concentration	500	25	15	5

Table S2 Theoretical concentration of pollutants in constructed wetland

Water Quality Index	COD（mg/L）	TN（mg/L）	NH_4^+-N（mg/L）	TP（mg/L）	PS NPs（mg/L）
Theoretical concentration	80	25	7	2	1

- *Table S3 Components of synthetic wastewater from constructed wetland*

Drugs	Concentration (mg/L)
Sodium acetate (CH_3COONa)	102.56
Urea ($CO(NH_2)_2$)	6.43
Ammonium sulfate ($(NH_4)_2SO_4$)	33.08
Potassium nitrate (KNO_3)	108.21
Potassium dihydrogen phosphate (KH_2PO_4)	8.78
Magnesium sulfate ($MgSO_4 \cdot 7H_2O$)	50.00
Ferrous sulfate ($FeSO_4 \cdot 7H_2O$)	3.50
Zinc sulfate ($ZnSO_4 \cdot 7H_2O$)	0.13
Sodium molybdate ($Na_2MoO_4 \cdot 2H_2O$)	0.03
Boric acid (H_3BO_3)	0.025
Copper sulfate ($CuSO_4 \cdot 5H_2O$)	0.03

Table S4 Test methods of water quality parameters

Water quality indicators	Measurement method
COD	Hash Micro Reflux Method
TP	Molybdenum Antimony Antiseptic Spectrophotometry
TN	Alkaline Potassium Persulfate Elimination UV Spectrophotometry
NH_4^+-N	Nano Reagent Spectrophotometry
NO_3^--N	Phenol Disulfonic Acid Spectrophotometry
NO_2^--N	N-(1-Naphthyl)-Ethylenediamine UV Spectrophotometry
PH	Mettler pH Meter

Table S5 Matrix enzyme activity assays

Enzyme indicators	Measurement methods
Dehydrogenase (enzyme)	Triphenyltetrazolium chloride colourimetric method
Urease	Nano reagent colourimetric method
Ammonia monooxygenase	Nitrite production rate method
Nitrite oxidase	Nitrite consumption rate method
Nitrate reductase	Nitrite generation rate method
Nitrite reductase	Nitrite consumption rate method
Neutral phosphatase	P-Nitrophenyl disodium phosphate colourimetric method

Table S6 - Elemental composition of BF and MBF (%)

Element	BF		Ca-MBF		Mn-MBF	
	Weight	Atom	Weight	Atom	Weight	Atom
C K	0.00	0.00	0.00	0.00	0.00	0.00
N K	0.00	0.00	0.00	0.00	0.00	0.00
O K	48.23	63.72	54.30	69.96	48.76	64.36
NaK	2.37	2.18	1.94	1.74	2.07	2.08
MgK	2.87	2.50	2.51	2.13	2.28	1.97
Al K	7.52	5.89	5.79	4.42	7.95	6.41
Si K	26.56	19.99	18.78	13.79	22.46	17.08
K K	1.68	0.91	1.51	0.80	1.53	0.83
Ca K	4.82	2.54	10.60	5.45	3.48	1.83
Ti K	0.48	0.21	0.45	0.19	0.37	0.16
MnK	0.00	0.00	0.00	0.00	6.92	3.70
Fe K	5.49	2.08	4.12	1.52	4.18	1.58

Table S7- Comparison of membrane hanging effect of BF and MBF

Sample	BF	Ca-MBF	Mn-MBF
Film Hanging Rate	98.36%	246.15%	260.12%
Residual Film Hanging Rate	1.97%	18.18%	30.09%

Information S1 Detection methods of water quality parameters

(1) Detection of Chemical Oxygen Demand (COD) by the Potassium Dichromate Method: Potassium dichromate ($K_2Cr_2O_7$) oxidizes reducing substances in water samples under acidic conditions, producing trivalent chromium ions (Cr^{3+}). The remaining hexavalent chromium ions (Cr^{6+}) are then reduced by ammonium ferrous sulfate ($NH_4Fe(SO_4)_2 \cdot 12H2O$). The COD value is calculated based on the amount of ammonium ferrous sulfate used.

(2) Detection of Ammonia Nitrogen (NH_3-N) by Colorimetric Method with Nessler's Reagent: Ammonia nitrogen in water reacts with Nessler's reagent (mercury iodide and potassium iodide) under alkaline conditions, forming a yellow complex. The absorbance is measured with a spectrophotometer and compared to a standard curve for quantification.

(3) Detection of Total Nitrogen (TN) by Alkaline Potassium Persulfate Digestion Ultraviolet Spectrophotometry: In alkaline conditions, organic and inorganic nitrogen in the water sample is oxidized to nitrate by potassium persulfate ($K_2S_2O_8$). The absorbance of nitrate ions is then measured by a UV spectrophotometer to determine the total nitrogen content.

(4) Detection of Total Phosphorus (TP) by Ammonium Molybdate Spectrophotometric Method: Total phosphorus in the water reacts with ammonium molybdate ($(NH_4)_6Mo_7O_{24} \cdot 4H_2O$) under acidic conditions to form a blue phosphor-molybdenum complex. Its absorbance is measured by a spectrophotometer and quantified by comparing it to a standard curve.
